# Demographic Characteristics and Medical Service Use of Failed Back Surgery Syndrome Patients at an Integrated Treatment Hospital Focusing on Complementary and Alternative Medicine: A Retrospective Review of Electronic Medical Records

**DOI:** 10.1155/2014/714389

**Published:** 2014-11-04

**Authors:** Hee Seung Choi, Eun Hya Chi, Me-riong Kim, Jaehoon Jung, Jinho Lee, Joon-Shik Shin, In-Hyuk Ha

**Affiliations:** Jaseng Spine and Joint Research Institute, Jaseng Medical Foundation, 858 Eonju-ro, Gangnam-gu, Seoul 135-896, Republic of Korea

## Abstract

*Objective*. To report the patient demographics and nonsurgical complementary and alternative medicine treatment used at a Korean medicine hospital for low back pain (LBP) and/or sciatica after surgery. *Methods*. Medical records of patients who visited a spine-specialized Korean medicine hospital at 2 separate sites for continuous or recurrent LBP or sciatica following back surgery were reviewed. The demographics, MRI and/or CT scans, and treatments were assessed. *Results*. Of the total 707 patients, 62% were male and the average age was 50.20 years. Ninety percent of patients presented with LBP and 67% with sciatica. Eighty-four percent were diagnosed with herniated nucleus pulposus at time of surgery. Of these patients, 70% had pain recurrence 6 months or later, but 19% experienced no relief or immediate aggravation of pain after surgery. Many patients selected traditional Korean medicine treatment as primary means of postsurgery care (47%). When time to pain recurrence was short or pain persisted after surgery, return of symptoms at the same disc level and side was frequent. *Conclusion*. An integrative treatment model focusing on Korean medicine and used in conjunction with radiological diagnostics and conventional medicine is currently used as a treatment option for patients with pain after lumbar spine surgery.

## 1. Introduction

Although low back pain (LBP) is considered to have a favorable natural prognosis, lumbar spine surgery is still frequently performed. In the US, the prevalence of lumbar fusion surgery has increased 220% from 1990 to 2001 [1], and 250,000 laminectomies are estimated to be conducted each year as of 2002 [2].

However, with the increase of surgery cases, the interest in failure rates is also on the rise. A 10- to 22-year follow-up study by Yorimitsu et al. on the outcomes of discectomy for lumbar herniated nucleus pulposus (HNP) reported 74.6% of patients had residual LBP and 12% required reoperation(s) [3]. In Korea, a retrospective cohort study using national health insurance data of 35,558 patients who received a first surgery for lumbar herniated intervertebral disc disease in 2003 reported that the cumulative reoperation rate at 5 years was 13.4% with half the reoperations performed in the first postoperative year [4]. Park and Kim reported the results of 186 patients seeking medical care due to persistent or aggravated pain after surgery, of whom 75% visited within 2 years of surgery and 35% required reoperations [5].

Other studies on the results of back surgery reported that adequate pain relief was not achieved in up to 30% of patients receiving a single lumbar segment operation [6] and that only 34% of reoperation patients had successful outcomes (at least 50% sustained relief of pain and satisfaction with the results), showing high postsurgery pain occurrence rates [7]. There are also studies reporting the difficulties of reoperation with success rates falling to 15% following a third back operation and around 5% after the fourth [8].

As more patients and practitioners are taking an interest in treatment for patients with pain after surgery, discussions continue to further demarcate the somewhat controversial term, failed back surgery syndrome (FBSS). The term FBSS is usually used to describe conditions presenting as recurrent or persisting LBP with or without sciatica after one or more lumbar surgeries [7], and numerous research results are being published on such conventional nonsurgical approaches as interventional pain procedures (e.g., epidural injections, intra-articular joint steroid injections, medial branch blocks, radiofrequency ablation, adhesiolysis, disc interventions, spinal cord stimulation, peripheral nerve stimulation, etc.), pharmacological management, interdisciplinary management with physical therapy and psychological therapy, and surgical revision in an attempt to determine optimally effective treatment methods [9].

Besides the conventional treatments listed above, there are various types of complementary and alternative medicine (CAM) originating in non-Western cultures or not yet accepted in mainstream medicine lacking scientific evidence. The UK National Health Service (NHS) website cites the US National Center for Complementary and Alternative Medicine (NCCAM) and states that when a treatment is used alongside conventional treatments to help a patient deal with a certain health condition, but not as an alternative, this use of treatment is called “complementary medicine” and when a treatment is used instead of conventional treatments with the intention of curing or treating a health condition, the use is called “alternative medicine” [10]. Traditional Chinese medicine (TCM) and traditional Korean medicine (TKM) treatment [11–15] which began in non-Western cultures are also regionally categorized and described as CAM and mainly consist of acupuncture, moxibustion, and herbal medicine and are widely used for the treatment of pain related to spinal disorders. We therefore report the comprehensive results of our review of the demographic characteristics of patients who visited 2 spine specialty Korean medicine hospitals, to provide basic data needed to establish the etiology of FBSS and describe treatment approaches employed in actual clinical settings.

## 2. Methods

Jaseng Hospital of Korean Medicine is a group of Korean medicine hospitals that specialize in spine disorders and is designated by the Korean Ministry of Health and Welfare as a spine specialty hospital, treating 900,000 cases of spinal disorders (e.g., nonspecific back pain, neck pain, intervertebral disc diseases, spondylolisthesis, compression fractures, etc.) each year. Treatment mainly focuses on HNP and spine-related pain patients prior to developing postsurgery syndrome, and the hospital employs a cohesive overall treatment approach using Korean medicine as the main modality and the imaging technology of conventional medicine and treatment, as needed, for emergency care. This retrospective multicenter study assesses the demographics of the patients who visited Jaseng Hospital of Korean Medicine in Seoul, Korea, and Bucheon Jaseng Hospital of Korean Medicine in Bucheon, Korea, from January to December, 2012, for low back pain or sciatica recurrence after surgery and the treatment received through electronic medical records.

Demographic characteristics included patients' sex, age, occupation, and medical history including hypertension/diabetes, site of pain, number of operations, recommendations for repeat surgery, duration of time from surgery to pain recurrence, duration of pain, limited lumbar range of motion (ROM) due to pain, pain intensity (NRS, Numeric Rating Scale), treatments received prior to current hospital visit, primary diagnosis for surgery as reported through patient recollection, mode of onset of pain recurrence, number of MRI/CT scans performed after surgery at study setting, number of MRI/CT scans performed at other clinics or hospitals, number of patients who received inpatient treatment, and average length of hospital stay.

The radiological readings of the MRI/CT scans of 288 patients evaluated by two experienced radiologists were used to categorize patients by radiological diagnosis at time of visit, level of HNP at time of spine surgery, method of surgery, and pain recurrence.

Lastly, the types and frequency of treatment, number of recipient patients, and average number of treatment sessions per patient were evaluated. The treatment mainly focused on traditional Korean medicine which encompasses herbal medicine, acupuncture, electroacupuncture, pharmacopuncture, bee venom pharmacopuncture, Chuna (Korean manual therapy), and physical therapy.

**Figure 1:**
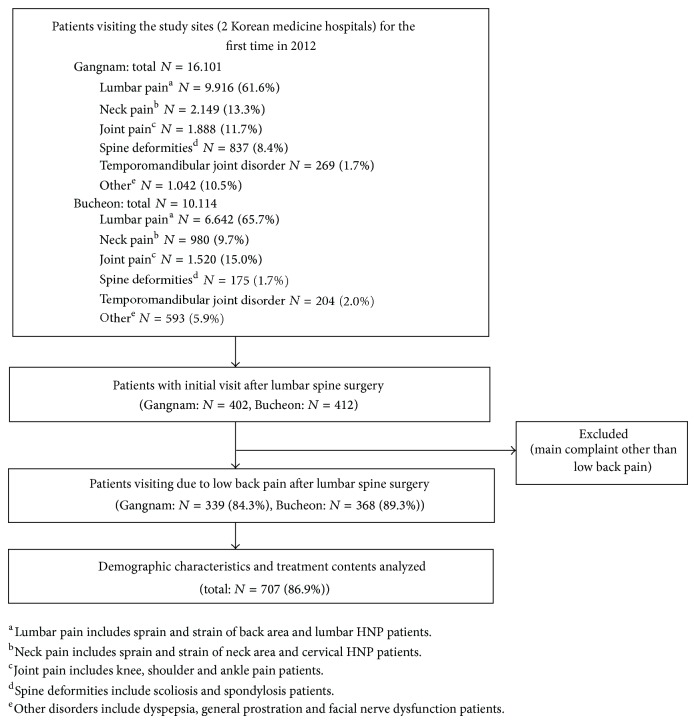
Flow diagram of the study.

This study was approved by the Institutional Review Board of Jaseng Hospital of Korean Medicine (SIRB2013-18) and adhered to research ethics (Figure 1).

## 3. Results

### 3.1. Patient Characteristics at Baseline (Table 1)

A total of 707 patients presented with recurrent LBP or sciatica after lumbar surgery at the two study sites in 2012, and 16% of these patients received an average 21.72 (±11.91) days of inpatient treatment. The average age was 50.20 (±15.15) yrs, and 62% were male.

Of the patients who visited the hospital due to LBP or sciatica following lumbar spine surgery, 90% had a chief complaint of LBP and 67% had sciatica (with or without LBP). Of subgroups classified by pain intensity, the majority (40%) had severe pain with an NRS of 7 or higher, and 31% of the total patients showed restricted ROM with pain.

Although 70% of patients had pain recurrence at 6 months or later after surgery, 22% were acute patients with pain recurring within a month. The average time to pain recurrence from surgery was 13.93 (±23.24) months.

The average number of surgeries was 1.16 (±0.41). Assessments for prior treatments revealed that 47% of patients, which was the highest proportion, made Jaseng Hospital of Korean Medicine their first choice of treatment, and 17% selected other providers of traditional Korean medicine, showing that many patients chose Korean medicine as their primary means of medical care for postsurgery pain. Sixteen percent had received recommendations for repeat surgery prior to their visit to the study site. The most commonly reported diagnosis for surgery was HNP which accounted for 84%.

### 3.2. Evaluation of Postsurgery MRI/CT Findings (Table 2)

The findings of the MRI/CT scans of 288 patients were recorded by experienced radiologists and assessed and categorized for study means. The vast majority were diagnosed with HNP (77%) and spinal stenosis (18%) through imaging. The most frequently operated disc level was L4/5 (58%). Analyses on correlations between time to pain recurrence after surgery and symptom recurrence at site of surgery and mode of onset of pain recurrence and symptom recurrence at site of surgery found that in cases with shorter periods to pain recurrence and in continuation of pain, same site and same disc level symptom recurrence was more likely to occur.

### 3.3. Types and Frequency of Treatment Used at the Two Hospitals of Korean Medicine (Table 3)

The treatment methods, frequency of treatment sessions, number of recipients, and average number of treatment sessions per patient were analyzed. The main treatment modality was TKM treatment including herbal medicine, acupuncture, electroacupuncture, pharmacopuncture (herbal extractions are used as stimulus for meridian points), bee venom pharmacopuncture, Chuna, and Korean medicine physical therapy based on traditional Korean medicine literature (such as Korean medicine herbal fomentation (steamed hot pack) therapy), and a small number of patients received conventional medicine treatment in addition to the main treatment. The records of herbal and conventional medicine intake were recorded in days and other treatments in number of sessions. The norm for outpatient treatment use was 1~2 times per week and that for inpatients was daily. Considering the relative difference in frequency, it can be inferred that inpatients received more intensive treatment in a compact amount of time than outpatients.

## 4. Discussion

Most patients visiting Korean medicine hospitals for postsurgery treatment in our study reported chronic conditions and intense pain. Also, a large proportion presented with more severe conditions with low back pain accompanied by sciatica. Furthermore, almost half the patients selected Jaseng Hospital of Korean Medicine or Bucheon Jaseng Hospital of Korean Medicine as their first choice for primary care, suggesting high expectation levels of TKM treatment in Korea. A practical approach using integrative TKM treatment was provided to the patients rather than a single treatment modality, and conventional medicine treatment was utilized as needed. On analysis of the MRI readings of 236 patients, the most frequent site of surgery was L4/L5.

McCulloch reported in a radiological research assessing pain recurrence the incidence of recurrence at the same disc level and ipsilateral side after surgery to be between 80% and 90% [16]. Other studies report that in symptom recurrence within 2 years, relapse is often at the operated disc level, while in cases that recur a few years later, relapse more often occurs at different levels [17–19]. Our results also demonstrate that in shorter periods of surgery to relapse, prevalence of HNP recurrence at the same level and same side increased, and in longer periods, the recurrence tendency was found to be at different disc levels. There was a higher chance for same level relapse in pain persisting after surgery with no significant period of relief as well.

The basic treatments received were acupuncture, herbal medicine, electroacupuncture, and pharmacopuncture. The results show that patients receiving inpatient care were offered more intensive treatment means than outpatients.

In an attempt to define the term FBSS, various standards have been suggested; one RCT specified FBSS as long-term continuance of chronic pain conditions [20], and some practitioners define FBSS as postlaminectomy syndrome which is also criticized as an inaccurate expression [2]. While some studies distinguish FBSS from recurrence, limiting the term to cases with persistent or exacerbated pain following surgery [21], some refer to a constellation of conditions covering all postsurgery pain continuance and relapse [7]. Suggested causes of FBSS range from canal stenosis, internal disc disruption, retained/recurrent disc disease, spondylolisthesis, synovial cyst, vascular claudication, instability, pseudomeningocele, pseudarthrosis, epidural fibrosis, degenerative disc, radiculopathy, facet syndrome, sacroiliac joint syndrome, and reflex sympathetic dystrophy to arachnoiditis, but debate continues on whether these conditions can be divided into surgical and nonsurgical causes [22]. Also, as not only the patient's congenital state, but also psychological and environmental factors need to be taken into account [23], Slipman purports that FBSS is not a specific single diagnosis, but a broad term referring to a group of disorders with a common feature of pain after surgery that should be understood as a syndrome with multiple possible etiologies [2].

Although the need for studies on the etiology of FBSS has been well explained and agreed on, only a limited number is currently available. We had initially considered reporting the demographics of a more clearly defined group of patients, but the large variation in previous definitions of FBSS prevented us from reaching a singular conclusion. The subjects included in our study were back surgery patients visiting the hospital for postoperative pain. While subjective pain levels requiring treatment may vary, all subjects visited the hospital for treatment purposes indicating a certain level of discomfort or pain. Therefore, the demographic characteristics of all patients with recurrent pain after surgery were collected and investigated to a broader extent than previous FBSS studies. The main mode of treatment was CAM focusing on TKM, and use of this integrative model of treatment based on TKM which is not yet well-known in the Americas and Europe was investigated and analyzed.

Pharmacopuncture is a relatively new form of TKM derived from a mixture of herbal medicine and acupuncture. The physical therapy referred to in our study is a Korean medicine physical therapy administered by Korean medicine doctors (KMDs) based on traditional Korean medicine literature (such as Korean medicine herbal fomentation (steamed hot pack) therapy). Therefore, pharmacopuncture and Korean medicine physical therapy fall under the category of CAM treatment as TKM modalities provided by KMDs. A small number of patients received conventional pharmacological therapy, injections, and nerve blocks through cooperative, integrative treatment with Western medicine doctors. However, the main objective of this study was not to prove the exclusive effects of CAM treatment, but to show the current use of integrative medicine at a CAM-oriented medical facility.

To address a few limitations of this study, first, in the process of interviewing the patients, we were dependent on the memory of the patients regarding information of their condition at time of surgery, so the accuracy of information could not be verified. Second, data collection focused on plain symptom listings with no definite differentiation between such chronic pain disorders as complex regional pain syndrome, myofascial pain syndrome, and fibromyalgia. Third, lack of evidence on the effectiveness of the separate modalities in TKM treatment restricted further analysis and interpretation.

The preference for CAM for spinal disorder treatment is increasing not only in Korea, but also in many other countries. According to a 2004 survey, 43% of peripheral neuropathy patients used CAM treatment for management of their symptoms, and the main reason for selecting CAM treatment was that many did not achieve adequate pain relief with standard conventional treatment. The most frequent CAM treatment methods used were acupuncture, chiropractic manipulation, herbal remedies, magnetic therapies, and vitamins [24].

The extent of CAM use is largely influenced by the medical system of each country. In Korea, equal licenses and independent practice rights for Korean medicine doctors and conventional medical doctors are guaranteed by the government, which is one of the reasons why Korean medicine could take on a leading role in this integrative treatment model. In this study, patients with recurrent pain after surgery received radiological diagnosis and requested conventional medicine treatments as necessary under the supervision of KMDs with Korean medicine treatment. However this could be deemed highly unusual in other cultural backgrounds.

## 5. Conclusions

This study showed how an integrative treatment model focused on Korean medicine treatment was used in patients with chronic and severe pain after back surgery. We hope that our study data may further understanding of FBSS patients who select Korean medicine treatment and be used as basic information in establishing an integrative treatment model for spinal disorders.

## Figures and Tables

**Table 1:** Patient characteristics at baseline (*N* = 707).

Sex, % male	441 (62%)	Mode of onset^f^	
Age, mean years (SD)	50.20 (15.15)	No specific cause	395 (59%)
Occupation		Continuous pain persisting or aggravated after surgery	137 (19%)
Unemployed^a^	163 (23%)	Sprain	122 (17%)
Office work^b^	154 (22%)	Trauma	24 (3%)
Service or retail industry^c^	67 (9%)	Numeric Rating Scale	
Manual labor^d^	32 (5%)	1~3	32 (4%)
Other	262 (37%)	4~6	166 (24%)
Unknown	29 (4%)	7~10	281 (40%)
Comorbidity		Missing values	228 (32%)
Hypertension (%)	134 (19%)	Prior treatment	
Diabetes mellitus (%)	65 (9%)	None	333 (47%)
Low back pain	629 (90%)	Pharmacologic therapy^g^	233 (33%)
Leg pain	471 (67%)	Physical therapy^h^	146 (21%)
Unilateral leg pain	344 (49%)	Traditional Korean medicine therapy^i^	117 (17%)
Bilateral leg pain	127 (18%)	Nerve block(s)^j^	101 (14%)
Number of surgeries	1.16 (0.41)	Percutaneous epidural neuroplasty	29 (4%)
Recommendation for repeat surgery	114 (16%)	Diagnosis at surgery^k^	
Limited lumbar range of motion with pain^e^	220 (31%)	Herniation of nucleus pulposus	590 (84%)
Length of current pain episode, mean months (SD)	13.93 (23.24)	Spinal stenosis	88 (13%)
Number of inpatients at study site (%)	111 (16%)	Vertebral fracture	12 (2%)
Average period of hospital stay	21.72 (11.91)	Others^l^	38 (5%)
Onset		Number of patients with MRI/CT scan(s) after surgery	484 (64%)
Acute (≤1 month)	158 (22%)	Number of patients with MRI/CT scan(s) at study site after surgery	288 (41%)
Subacute (1–6 months)	51 (8%)	Number of patients with MRI/CT scan(s) after surgery at other clinics or hospitals	196 (28%)
Chronic (>6 months)	498 (70%)	Average number of MRI/CT scans, mean(SD)^m^	0.85 (0.96)
		Period to pain recurrence in patients with recurrent low back pain after surgery as diagnosed through positive MRI scans, mean months (SD)	50.67 (66.46)

SD = standard deviation.

^a^Housewife/student/retired.

^b^Office worker/public servant/professional practitioner.

^c^Self-employed/service or retail industry worker.

^d^Agriculture, forestry, fishery, or mining industry worker/equipment mechanic or machinery operator/professional soldier.

^e^Number of patients with positive physical examination findings including lumbar flexion, extension, right lateral bending, and left lateral bending.

^f^14 patients were excluded due to pain complaints of nonmedical origin.

^g^Muscle relaxation, antineuropathic drugs, nonsteroidal anti-inflammation drugs, and opioids.

^h^Physiotherapy, occupational therapy, and exercise therapy.

^i^Acupuncture, Chuna, pharmacopuncture, bee venom pharmacopuncture, and herbal medicine.

^j^Caudal epidural blocks and nerve root blocks. ^k^Diagnosis for surgery as recalled by patients.

^l^Any self-reported spinal disorders, including spondylolisthesis, spinal abscess, spinal meningiomas, myelitis, scoliosis, and viral encephalomyelitis.

^m^Average number of MRI/CT scans performed after surgery at any site.

**Table 2:** Evaluation of postsurgery MRI or CT findings (*N* = 288)^a^.

Radiological findings of MRI/CT scans	
Herniation of nucleus pulposus	223 (77%)
Spinal stenosis	53 (18%)
Epidural fibrosis	7 (2%)
Spondylolisthesis	7 (2%)
Vertebral fracture	3 (1%)
Other^b^	10 (3%)
Operated disc levels	
L1/2	6 (2%)
L2/3	10 (3%)
L3/4	40 (14%)
L4/5	169 (58%)
L5/S1	96 (33%)
Types of surgery	
Laminectomy	206 (72%)
Fusion	28 (9%)
Other^c^	13 (5%)
Recurrence at operated disc level	
Same level, ipsilateral side	95 (33%)
Same level, contralateral side	30 (10%)
Different level	98 (34%)
Undefined^d^	65 (23%)

^a^Patients whose MRI/CT scan readings were interpreted by radiologists at the study site. Duplication was allowed in all records except relapse with regard to site of surgery. Due to inability to radiologically identify any evident sign of surgery, the surgical area and types of surgery were excluded in 52 patients.

^b^Facet arthrosis, retrolisthesis, spondylitis, and tumors.

^c^Vertebroplasty, disc replacement, and facetectomy.

^d^Cases of pain where no definite relation could be identified between pain and intervertebral disc and cases where the site of surgery could not be identified radiologically.

**Table 3:** Types and frequency of treatment used at the two hospitals of Korean medicine.

	Total (*N* = 702)			Outpatients (*N* = 591)			Inpatients (*N* = 111)		
Average number of sessions (SD)^a^	15.75 (18.17)			12.26 (15.88)			34.85 (18.17)		
	Total sessions	Number of patients	Mean number of sessions per patient	Total sessions	Number of patients	Mean number of sessions per patient	Total sessions	Number of patients	Mean number of sessions per patient
Acupuncture^b^	9269	615 (88%)	15	4935	505 (85%)	10	4334	110 (99%)	39
Herbal medicine^c^	43229	511 (73%)	85	36094	416 (70%)	87	7135	95 (86%)	75
Electroacupuncture^b^	6428	492 (70%)	13	3559	391 (66%)	9	2829	101 (91%)	28
Pharmacopuncture^b^	5139	489 (69%)	11	2768	381 (64%)	7	2371	108 (97%)	22
Bee venom pharmacopuncture^b^	3579	269 (38%)	13	2307	195 (33%)	12	1272	74 (67%)	17
Chuna^b^	2696	237 (34%)	11	1795	176 (30%)	10	901	61 (55%)	15
Korean medicine physical therapy^b^	3226	280 (40%)	12	1098	173 (29%)	6	2128	107 (96%)	20
Pharmacological therapy^c^	1008	53 (8%)	19	566	26 (5%)	21	442	27 (24%)	16
Injections^d^	72	26 (4%)	3	17	11 (2%)	1.5	55	15 (14%)	3.7
Nerve blocks^d^	80	37 (6%)	2	62	13 (2%)	1.8	56	24 (22%)	2.3

Patients may have received multiple treatment interventions during each session; therefore, the total number of patients is not equal to the total number of patients who received treatment.

^a^One day of hospital stay was considered equivalent to 1 hospital visit.

^b^Frequency of treatment was set at once or twice a week for outpatient treatment and daily for inpatient treatment.

^c^Days of medication intake.

^d^Applied as necessary.
